# A glycine receptor is involved in the organization of swimming movements in an invertebrate chordate

**DOI:** 10.1186/1471-2202-11-6

**Published:** 2010-01-19

**Authors:** Atsuo Nishino, Yasushi Okamura, Stefania Piscopo, Euan R Brown

**Affiliations:** 1 Department of Biological Sciences, Graduate School of Science, Osaka University, Machikaneyama 1-1, Toyonaka, Osaka 560-0043, Japan; 2 Okazaki Institute for Integrative Bioscience, National Institutes of Natural Sciences, Higashiyama 5-1, Myodaiji, Okazaki, Aichi 444-8787, Japan; 3 Department of Integrative Physiology, Graduate School of Medicine, Osaka University, Yamada-Oka 2-2, Suita, Osaka, 565-0871, Japan; 4 Laboratorio di Fisiologia Animale ed Evoluzione, Stazione Zoologica Anton Dohrn, Villa Comunale, 80121 Napoli, Italia

## Abstract

**Background:**

Rhythmic motor patterns for locomotion in vertebrates are generated in spinal cord neural networks known as spinal Central Pattern Generators (CPGs). A key element in pattern generation is the role of glycinergic synaptic transmission by interneurons that cross the cord midline and inhibit contralaterally-located excitatory neurons. The glycinergic inhibitory drive permits alternating and precisely timed motor output during locomotion such as walking or swimming. To understand better the evolution of this system we examined the physiology of the neural network controlling swimming in an invertebrate chordate relative of vertebrates, the ascidian larva *Ciona intestinalis*.

**Results:**

A reduced preparation of the larva consisting of nerve cord and motor ganglion generates alternating swimming movements. Pharmacological and genetic manipulation of glycine receptors shows that they are implicated in the control of these locomotory movements. Morphological molecular techniques and heterologous expression experiments revealed that glycine receptors are inhibitory and are present on both motoneurones and locomotory muscle while putative glycinergic interneurons were identified in the nerve cord by labeling with an anti-glycine antibody.

**Conclusions:**

In *Ciona intestinalis*, glycine receptors, glycinergic transmission and putative glycinergic interneurons, have a key role in coordinating swimming movements through a simple CPG that is present in the motor ganglion and nerve cord. Thus, the strong association between glycine receptors and vertebrate locomotory networks may now be extended to include the phylum chordata. The results suggest that the basic network for 'spinal-like' locomotion is likely to have existed in the common ancestor of extant chordates some 650 M years ago.

## Background

**Figure 1:**
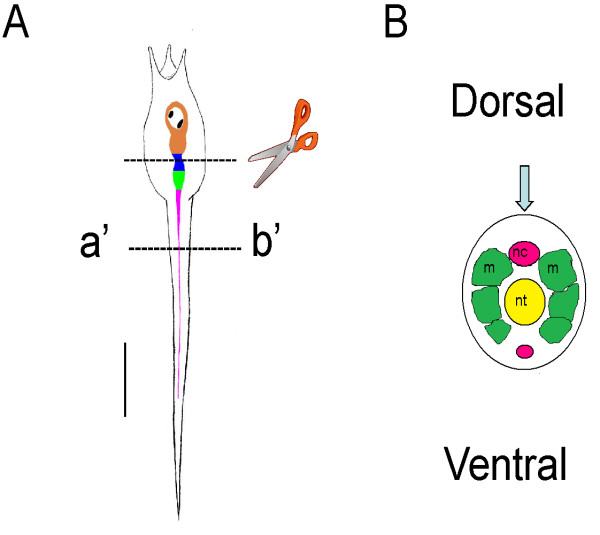
**Simplified diagrams showing *Ciona intestinalis *larval body plan and 'central nervous system'**. (*A*) Larval body plan showing the central nervous system divisions. Orange, brain vesicle (BV) containing photoreceptive ocellus and a gravity sensing otolith (black spots). Green, presumptive motor ganglion, known variously as the visceral, trunk or tail ganglion. Pink, nerve cord (NC). The upper dotted line shown the region of the section made when preparing 'headless' larvae. The lower section on the line a'-b' shows the region of the cross section shown diagrammatically in *B*. (*B*) Diagram showing the main features of the nerve cord (pink), muscle (green) at the cross-section at the line a'-b'. The blue arrow shows the angle of view in *A*. This diagram is used in the following figures to show the angle of view in the micrographs. Scale bar in *A*, 100 μm.

Ascidians (urochordates) and vertebrates are close relatives, and shared a common chordate ancestor around 650 million years ago [1]. For this reason, ascidians have been studied for nearly 150 years as a means to uncover the origin and evolution of their more complex vertebrate relatives [2]. With the recent sequencing of the genome of *Ciona intestinalis *[3], there has been an increase in interest in ascidians as useful models to study the evolution of chordates. In addition, anatomical traits and gene expression patterns have predicted 'homologies' between the ascidian larval nervous system and the vertebrate central nervous systems (CNS) [4,5] that may indicate deeper homology at the level of the neural networks to generate similar functions. The larval nervous system is rather simple (shown diagrammatically in Figure 1A) when compared to that of vertebrates and consists of some 80-100 neurons [6], divided into two or three main sub-divisions; a brain vesicle (BV) that contains a photoreceptive ocellus and a gravity sensing otolith; a presumptive motor ganglion (MG) known variously as the visceral, trunk or tail ganglion; and the nerve cord (NC). (Recently the use of 'visceral ganglion' as the common term for the motor ganglion, has been called into question [5,7], as there are no *viscera *in the larva. The alternatives suggested are 'trunk' [5] or 'tail' ganglion [7] though here we use *motor ganglion *(MG) as a more appropriate term). Although these nervous system divisions express *Hox *genes in a way that suggest these structures represent the equivalent of the forebrain, hindbrain and spinal cord [4,8], another analysis suggests that there are two divisions where the BV represents the forebrain, and the MG and NC the spinal cord [5].

**Figure 2:**
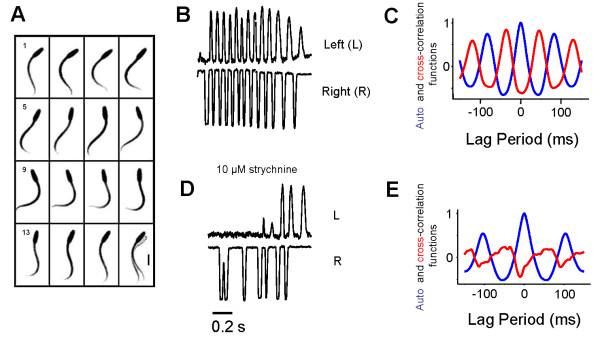
**Swimming patterns and pharmacological effects in the larva of the ascidian *Ciona intestinalis***. (*A*) Montage of images from high speed video of swimming at 6 ms intervals showing alternating swimming movements and larval progression (in the last frame the final position overlays the first position (grey). Sequence runs from left to right and top to bottom. Scale bar in frame 13, 100 μm. (*B*) Traces showing strict left/right (L/R) alternation of tail movements during swimming strokes in a tethered decapitated larva in 1 mM L-glutamate. (*C*) Phase relation of the autocorrelation on the left side (blue) with the cross correlation (red) between left and right sides. (*D*) The same larvae showing loss of strict L/R alternation in the presence of strychnine. (*E*) Phase relation from the same larvae as in (*C*) showing a strong autocorrelation on the left side and no positive correlation with the right side (red) in the presence of strychnine.

We examined the neural control of swimming in the larva of *C. intestinalis *to understand if the similarities in anatomy and gene expression patterns reflect similar physiological mechanisms for the control of locomotion in these two chordate subphyla (vertebrates and urochordates). Although ascidians have a sessile adult form, the tadpole-like larva swims with rapidly alternating tail beats that superficially resemble vertebrate swimming (Figure 2A). Intracellular recording from muscle cells from two distantly spaced electrodes shows however near-simultaneous activation of the muscles on one side of the tail during swimming strokes [9]. This means that the traveling wave that is generated is due to the interaction of the muscle, notochord and resistive forces with the surrounding fluid and not because of sequential activation of segmented muscle cells as in vertebrates. In vertebrates, alternating activity during swimming is generated in the spinal cord by the activation of both excitatory glutamatergic interneurons and inhibitory glycinergic commissural interneurons that periodically inhibit contralaterally located motoneurons [10]. Thus, the travelling wave during swimming is produced by sequential activation of coupled spinal networks acting on segmented muscle. Despite these fundamental differences, here we find that the contribution of glycinergic inhibitory transmission is a common feature in both vertebrate and non-vertebrate chordate locomotory control.

## Methods

### Animals

*Ciona intestinalis *adults were collected in the bay of Naples by the fishing service of the Stazione Zoologica and also from Nishiura port and Issiki port in Gamagori (Aichi, Japan). Adults were kept in the tanks of the Stazione Zoologica and Okazaki Institute for Integrative Bioscience. For physiological experiments in Naples, gametes were collected from the gonoducts of several animals and used for *in vitro *fertilization. Fertilized eggs were then raised in incubators in filtered seawater at 17°C (under these conditions larvae hatch at around 19.5-20.5 hr). In Okazaki, developing embryos were reared at 18°C in artificial seawater (Jamarin U artificial seawater, Jamarin Laboratory, Japan) (larvae hatched at around 17 hours post-fertilization; hpf). Hatched larvae were then transferred to experimental chambers for recording at 18-20°C. Larvae were partially immobilized by pinning them down by the tail or head with *Opuntia *glochids to Sylgard coated Petri dishes. In some cases, the 'head' was transected between the motor ganglion (MG) and brain vesicle (BV) leaving only the MG and tail intact (Figure 1A). Otherwise, after pinning down the head, the tunic was removed from the tail with fine forceps. This procedure allowed better penetration of the drugs.

### Motion analysis

Larval movements were recorded either with a high speed video (FAST-CAM Rabbit mini 2, PHOTORON, Tokyo, Japan) at 400-500 fps or were monitored in real time using a novel dual emission detection unit consisting of two photomultipliers (PMTs, Cairn Research Ltd, Faversham, Kent, UK) adapted to enable the simultaneous measurement of density from two regions of the field of view. In brief, this involved producing two separate images by passing the emitted light through a broad- band 50% split dichroic mirror. 50 % of the light was sent to photomultiplier one and the rest of the light filtered through a 600 nm band pass dichroic mirror that passed all light under 600 nm to photomultiplier two. >600 nm red light was passed to a video camera (Sony CCD CC-7) that had been modified to be sensitive to red light (IR filter was removed). The rectangular apertures of two variable shutters positioned in each of the two light paths leading to the PMTs, were visualized on the image produced by the video camera by backlighting each shutter aperture with light produced by two red (635 nm) light emitting diodes. The 'regions of interest' formed by the shutter apertures were the points of maximum left and right excursion of the tail or head of the tethered larva. The experiment was setup by manually selecting the two areas of interest. The outputs of the two photomultipliers, which were set to a moderate gain, were digitized as described below.

For video analysis, captured images were recorded on videotape, digitized through a Digital Handycam (Sony, Japan) or an A/D convertible video board mounted on a PC. Two 'regions of interest' were selected either from the video or with the dual photomultiplier system from the screen, and the density changes measured to detect alternate movements of the larval tail. Frames were analyzed using software (NIH Image, ver. 1.6), and photomultiplier records were digitized and stored using a Digidata 1200 data acquisition system and finally analyzed using Clampfit software (ver. 9.0) (both from Molecular Devices, formerly Axon Instruments, Union City, CA, USA). The locomotor period was calculated by carrying out an autocorrelation analysis on the left side of each data trace and measuring the time between peaks (eg, Figure 2). The contralateral phase relation was determined by dividing the time difference between the peak of the left side autocorrelation and the left/right cross-correlation trace by the locomotory period (Figure 2).

### Drugs and solutions

Drugs, compounds and solutions were added or continuously perfused over the preparation at a rate of 8 mL/min. Picrotoxin was purchased from Tocris but other drugs and compounds were from Sigma unless otherwise specified. The drugs were prediluted from concentrated stock solutions and added directly to the bath or to the superfusate at the final concentrations indicated. Picrotoxin and strychnine were dissolved as concentrated stocks (1-10 mM) in absolute ethanol. During these experiments, penetration of drugs through the intact tunic was very limited (100 μM strychnine or picrotoxin having no effect). Removal of the outer tunic or section of the head gave reliable penetration at lower concentrations.

### Immunocytochemistry

*Ciona intestinalis *larvae were fixed by immersion in 0.1% glutaraldehyde and 4% formaldehyde (as paraformaldehyde) in phosphate-buffered saline (PBS) at pH 7.0-7.4 diluted 50% in seawater. Samples (15 ml) were microwaved at 550 W for 5 s, then washed in three changes of 0.1 M PBS/0.25% Triton-X for 10-15 min each. Larvae were mixed frequently to obtain uniform permeabilization. They were then washed five times for 10 min each in 0.2% Triton-X in PBS before blocking in 1 mg/ml goat serum in 0.2% Triton-X PBS, all day or overnight at 4°C. They were then incubated in a primary antibody, rabbit anti-glycine polyclonal antiserum [11] (AB139, Millipore Bioscience Research Reagents [formerly Chemicon], Temecula, CA, USA) in 0.2% Triton-X PBS for 48 h at 4°C at a dilution of 1:250, followed by six washes in 0.2% Triton-X PBS. The larvae were then incubated in Alexafluor-594 conjugated goat anti-rabbit secondary antibody (Invitrogen Corporation, Carlsbad, California, USA) at a dilution of 1:200 in 0.2% Triton-X PBS for 3 h at 4°C and washed five times in PBS. Finally, larvae were mounted in Vectashield beneath 00 coverglass, viewed either with × 25/0.8 or ×40/1.3 Plan Neofluar objectives and image stacks collected using a confocal microscope (LSM510, Zeiss). Images from the LSM510 software were prepared in Adobe Photoshop.

### Molecular Biology

For isolation of the full-length cDNA of the glycine receptor (*Ci-GlyR*) candidate from *C. intestinalis*, we utilized the dataset from a collection of ion channel sequences [12] and online databases for the genome sequence (Joint Genome Institute, *C. intestinalis *ver. 1, http://genome.jgi-psf.org/ciona4/ciona4.home.html, and ver. 2, http://genome.jgi-psf.org/Cioin2/Cioin2.home.html). We predicted the single cognate (ver. 1 gene model "ci0100137620"; ver. 2 gene model "gw1.106.72.1") by online homology search and obtained the full-length cDNA for that by performing 5' and 3' rapid amplification of cDNA ends (RACE) using a kit (GeneRacer, Invitrogen) and primers; outer reverse for 5'-RACE: GATGGATGAAGAGCAACCGCATCT, inner reverse for 5'-RACE: GAACCCAGTGAACGCCATGCGTT, outer forward for 3'-RACE: ACACACACCGAATCCACTGAGGAA, inner forward for 3'-RACE: GGTATGACCAAAGGATACGACCTCAT. Template cDNA was prepared from a total RNA pool from hundreds of hatched larvae (at about 18 hpf, 18°C). We cloned and sequenced multiple fragments for both ends and compared the results with that predicted from the genomic databases. We did not find any splice variants in those RACE fragments. The fragment pair with an identified open reading frame was combined via constructive PCR, and the obtained full-length cDNA was cloned into pCR4-TOPO (Invitrogen). The whole cDNA sequence of *Ci-GlyR *was confirmed by reading both strands.

The 2.5 kb upstream sequence from the predicted first Met of *Ci-GlyR *gene was isolated by genomic PCR using a pair of primers; GlyR-up-2500 (*Sal*I): GGCGGAGCTCTGCGTCGACGCAAGGCACATTA, GlyR-up-0 (*BamH*I): GGTGGATCCATCTTTAGAAGATATTGAATAAATGA. The isolated fragment was digested by *Sal*I and *BamH*I and cloned into *Sal*I-*BamH*I site of a expression vector, pEGFPΔCMV, derived from pEGFP-N1 (Clontech) with a deletion of the original CMV promoter (*Ase*I-*Nhe*I) (a gift from S. Yamada and H. Takahashi at the National Institute of Basic Biology, Okazaki, Japan). This *P_Ci-GlyR_EGFP *construct (10 μg/ml circular plasmid form dissolved in 200 mM KCl) was manually injected into *C. intestinalis *fertilized eggs as described previously [13].

### Whole-mount in situ hybridization

The coding sequence and the full-length of cDNA were linearized, and the sense and antisense probes were prepared using digoxigenin-labeling mix (Roche) and T3/T7 RNA polymerase by standard methods. The synthesized RNA probes were fragmented to ~300 bp. Whole-mount in situ hybridization was performed according to the protocol described previously [14] with minor modifications. The embryos or larvae at several stages were fixed with 4% paraformaldehyde in 0.5 M NaCl and 0.1 M MOPS (pH 7.5) overnight at 4°C. The specimens were stored at -20°C in 80% ethanol. They were rehydrated in PBST (phosphate-buffered saline containing 0.1% Tween-20) and partially digested with Proteinase K in PBST (3 μg/ml for embryos; 6 μg/ml for larvae) for 40 min at 37°C. After washes with PBST, the specimens were postfixed with 4% paraformaldehyde in PBST for 1 hr at room temperature (pH 7.5). After thorough washes with PBST, they were pre-hybridized with hybridization buffer, containing 50% formamide, 5× SSC, 5× Denhart's Soln, 0.1 mg/ml (for embryos) or 1 mg/ml (for larvae) yeast tRNA, and 0.1% Tween-20 for more than 2 hr at 50°C. Then, they were soaked in hybridization buffer containing probes (0.1-0.3 μg/ml for embryos, 0.01-0.05 μg/ml for larvae) for two days at 50°C. The hybridized embryos/larvae were thoroughly washed with 2× SSC, 50% formamide, 0.1% Tween-20 at 50°C for 30 min, and then the excess probes were digested with RNase A (20 μg/ml in a reaction solution containing 0.5 M NaCl, 10 mM Tris-Cl (pH 8.0), 5 mM EDTA, 0.1% Tween-20) for 30 min at 37°C. After further washes once with 2× SSC, 50% formamide, 0.1% Tween-20 at 50°C for 20 min, twice with 0.5× SSC, 50% formamide, 0.1% Tween-20 at 50°C for 30 min, the samples were soaked in blocking solution (0.5% (w/v) blocking reagent [Roche] in PBST), and then in the 1/2000 alkaline phosphatase (AP)-conjugated anti-digoxigenin (anti-DIG) antibody (Fab fragment, Roche) in the blocking solution (1 day at 4°C). After extensive washes of non-reacted antibodies with PBST, the signal was detected with standard NBT/BCIP staining for AP. After a few washes with PBST, the stained specimens were observed under the light microscope. No evident signal was found with the sense probe for *Ci-GlyR *(not shown); no difference was found between the antisense probes from the coding sequence only and from the full-length of *Ci-GlyR *cDNA.

For double labeling, the *Ci-ChAT *gene was used as reference, whose coding sequence was amplified by PCR using primers (ChAT-codeF: CATGCCTGGTGCACTACATCAGAA, ChAT-codeR: CGGATATTTAAGAAATGGTGATATTGT) from the larval cDNA, and subcloned into pCR4-TOPO. Fluorescein-labeled (Fluo-labeled) antisense probe was prepared from the cloned *Ci-ChAT *(Roche), and it was hybridized together with DIG-labeled *Ci-GlyR *probe at the same time. After blocking as in the procedure above, 1:2000 anti-DIG-AP and 1:2000 anti-Fluo-POD, or in some cases the same concentrations of anti-DIG-POD and anti-Fluo-AP (all Fab fragments from Roche), antibodies in blocking solution were treated at 4°C overnight. After thorough washes with PBST (1 hr, more than 4 times, RT), and two times more with PBS, the tyramide signal amplification (TSA) reaction was carried out (Invitrogen; HRP-goat anti-mouse IgG and Alexafluor-647 tyramides) for 1 hr at 4°C and then 4 hr at RT. After several washes with PBST and then with a buffer containing 0.1 M Tris-HCl (pH 8.0), 0.1 M NaCl, 10 mM MgCl_2_, coloring reaction were performed on AP labels by Fast-Red and HNPP (Roche) for 1.5-4 hr at RT. The specimens were washed with PBST and post-fixed with 4% paraformaldehyde in PBST for 1 hr at RT. The nuclei were visualized with 1 μM SYTOX Green in PBST for more than 20 min at RT. The double- or triple-stained specimens were observed in PBS under the confocal microscopy through appropriate filter sets for green (optional), red and infrared light detection. The images were shown with pseudo-colors and processed in Adobe Photoshop.

### Electrophysiology on *Xenopus *oocytes

The coding sequence of Ci-GlyR was amplified from the full-length cDNA inserts via PCR using primers; Ci-GlyR_forward (*Sal*I): GATCTGTCGACCGCCACCATGCAGTCGCAGTATAATG, Ci-GlyR_reverse (*Not*I): CTAGAGTCGCGGCCGCTTTAAACAGACAACACATAACC. The amplified fragments were subcloned into *Xho*I-*Not*I site of pSD64-TF (kindly provided by Dr. T. Snutch, Univ. of British Columbia). After confirmation of the sequence and linearization with *Xba*I, cRNA was synthesized using the procedures provided by a commercially manufactured kit (mMessage mMachine SP6, Ambion). *Xenopus laevis *oocytes were prepared as described [15], and experiments were performed according to the guidelines of the Animal Care Committee of the National Institute for Physiological Sciences, Okazaki, Japan. Frogs were anaesthetized by immersion in water containing 0.15% tricaine. Isolated oocytes were defolliculated with collagenase (1 mg/ml, S-1, Nitta Gelatin), and 50 nl of 0.02-0.05 μg/ml cRNA solution was injected. After incubation at 18°C for 2-5 days in ND96++ solution (96 NaCl, 2 KCl, 2 CaCl_2_, 1.8 MgCl_2_, 5 Hepes (pH 7.5), 550 μg/ml sodium pyruvate, 100 μg/ml gentamicin; unit = mM if abbreviated), oocytes were placed in a chamber of approximately 150 μl volume. Macroscopic glycine-induced currents were recorded under two-electrode voltage clamp using a 'bath-clamp' amplifier (OC-725C-HV, Warner Instruments). Data acquisition and analysis were done on a Macintosh computer using an ITC-16 AD/DA converter and Pulse software (HEKA Electronik). Intracellular microelectrodes were filled with 3 M KCl with resistances between 0.2-0.7 MΩ in the standard bath solution, ND96 (96 NaCl, 2 KCl, 2 CaCl_2_, 1.8 MgCl_2_, 5 Hepes [pH 7.5]). Oocytes were clamped at -50 mV, and ~2.5 μl of agonist solution was added directly to the bath. The agonists were sometimes removed by perfusion of the external solutions. To determine the ion selectivity from the shift of reversal potential, different bath solutions and a ramp pulse protocol were used. The digitized traces were analyzed offline using Pulse/Pulsefit (HEKA), Igor Pro (Wavematrix), and Excel (Microsoft).

### Morpholino injection

To suppress the expression of Ci-GlyR, we utilized a morpholino-oligo (MO) designed against the *Ci-GlyR *(GeneTools). The sequence of antisense(+) MO was GCAGACATTATACTGCGACTGCATC, while that of the control MO with 5 nucleotide-mismatches(-) was GCAcACATTATAgTcCGAgTGgATC. The MO was injected manually into intact fertilized eggs without dechorionation as above in the section **Molecular Biology **(ref. [13]), at concentrations of 0.5-0.6 mM in 200 mM KCl.

### Molecular phylogeny

To determine the phylogenetic position of Ci-GlyR, the predicted polypeptide sequence was aligned to the known GlyR subunits from other organisms by ClustalW. After excluding gaps from the alignment, phylogenetic relationships were examined by neighbor-joining methods using MEGA4 [16].

### Accession numbers

The sequence of *Ci-GlyR *was deposited in DDBJ/EMBL/GenBank under accession no: AB437088.

### Statistics

Statistical significance was tested where appropriate using Student's paired or unpaired tests.

## Results

### Physiology and pharmacology of swimming

*Ciona *larvae swim with alternating beats of the tail (Figure 2A, B, Additional file 1). To analyze this tightly alternating activity, movements of the tail were measured photometrically and the results subjected to auto- and cross-correlation analysis. Intact tethered larvae swam with a frequency of activity on the left side of 12.24 ± 1.0 Hz (n = 5) and with a left-right phase of 0.54 ± 0.01. To determine the role of the MG in pattern generation, we microdissected away the 'head' region of living larvae containing otolith and ocellus, leaving only the MG and tail intact. Such 'headless' larvae were immobile (Additional File 1). Addition of L-glutamate (100 μM) induced 'swimming' that consisted of highly precise alternating tail movements with an identical left-right phase to that seen in intact larvae (0.54 ± 0.02 [mean ± SEM], n = 5; Additional File 1) though at a significantly higher frequency 24.4 ± 1.0 Hz (P < .001 Student's paired t- test). When 10 μM L-glutamate was used the frequency of tail activity on one side was 11.68 ± 1.3 Hz with a left-right phase of 0.54 ± 0.006 (n = 5), not significantly different from the swimming rate of intact larvae.

To establish if inhibitory synaptic transmission is involved in the production of appropriate swimming patterns, we examined the effects of blocking glycinergic or GABAergic transmission by adding strychnine or picrotoxin to skinned or microdissected larvae (where swimming had previously been activated with 10 μM L-glutamate). There was a loss of strict alternation of L/R swimming in the presence of strychnine (Figure 2B, C, D, E; 24/30 larvae). As can be seen in the figures, a regular pattern of activity is generated on each side that may switch occasionally from one side to the other, but the strict sequence of alternation (L/R/L/R/L/R) seen in control larvae breaks down (e.g. L/L/L/R/R/R) (Figure 2 compare 2B and 2C). A more detailed analysis was carried out with the photomultipler system on six larvae and the results subjected to cross-correlation analysis (examples are shown in Figure 2C, D). The frequency of activity on the left side was 12.5 ± 1.2 Hz (not significantly different from the control), and a reliable measure of the left-right phase was not possible, as there was no cross-correlated right phase peak (n = 6). As previously reported [17], picrotoxin (Additional File 2B; 20/25 larvae) extended the swimming period and increased the frequency of activity on one side. We subjected five larvae to photometric analysis and the results (Additional File 2C, D) show an increase in frequency to 14 ± 1.5 Hz, and no disruption of the left-right phase (0.53 ± 0.005, n = 5).

### Location of putative glycinergic interneurons

**Figure 3:**
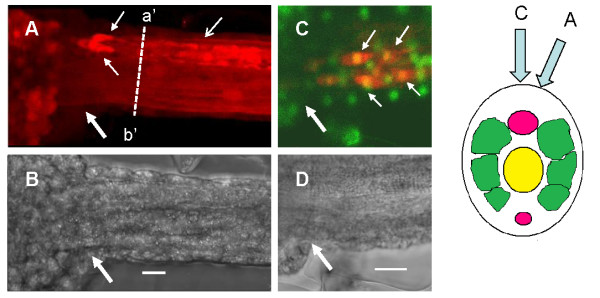
**Glycine immunocytochemistry at the junction of the 'tail' and 'head' region of *Ciona *larvae**. (*A*) Fluorescent image of the junction of tail and 'head' showing the location of glycine positive elements (red). Note the two clearly discernable glycine-positive cells (small arrows) in the nerve cord. (*B*) Brightfield image of the same area. (*C*) Fluorescent image of a similar area as in A showing dual staining of glycine positive material (red) and nuclei (green). In this case, at least four glycine positive elements may be identified as being cellular (yellow co-label). Large arrows indicate the junction between the 'head' and tail of the larva. Scale bars = 10 μm.

To locate possible glycinergic interneurons, we carried out immunocytochemistry using an anti-glycine antibody. A strong positive signal was found in two to four paired cells located in the nerve cord in a region posterior to the MG (Figure 3, and Additional File 3, representative of 20 larvae). Axons and apparent nerve terminals from these cells ran in a posterior direction for around 200 μm. Double staining with the nuclear dye CY-green, revealed denser parts of these positively staining elements to be 'cell bodies' and thus indicated that the more distal parts may be synapse-like terminals on or near the motoneuron axons and the upper two of the three rows of muscle cells on each side. The dimensions of the somata of these cells were around 4-5 μm fitting well in the lower range given for the dimensions of *Ciona *neurones of 5-10 μm [18], and unlike the motoneurones (to be described later), processes were not seen to leave the nerve cord.

### Glycine receptor

**Figure 4:**
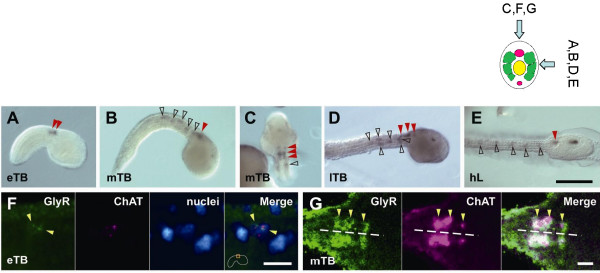
**Expression pattern of *Ci-GlyR***. (*A-E*) Whole-mount in situ hybridization of the *Ci-GlyR *antisense probe during larval development. The signal in the nervous system is marked with red arrowheads, and that in muscle cells is with open black arrowheads. eTB, early tailbud stage; mTB, mid-tailbud stage; lTB, late tailbud stage; hL, hatched larva stage. Scale bar on *E *= 100 μm for *A-E*. (*F-G*). Pseudo-color images of fluorescently detected *Ci-GlyR *and *Ci-ChAT *expression. (*F*) In early tailbud (eTB), *Ci-GlyR *(green, yellow arrowheads) and *Ci-ChAT *(magenta) expression can be detected in the cytoplasm of single neuronal cells (determined by labeling the cell nuclei (blue) with SYTOX Green), although it should be noted that the expression patterns do not overlap until the mid-tailbud stage (though nuclear staining show that the signals are in the same cell). Scale bar = 10 μm. Beside the scale bar, the location of the depicted region is outlined with an orange rectangle. (*G*) *Ci-GlyR *and *Ci-ChAT *transcripts are co-localized at the mid tailbud stage (mTB). The dashed line indicates the midline of the nerve cord. Scale bar = 10 μm.

**Figure 5:**
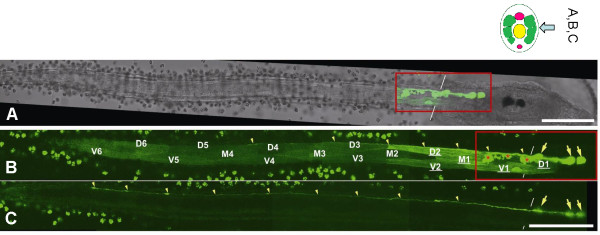
**(*A-C*) Expression of enhanced GFP under the control of a 2.5 kb 5'-flanking sequence of the *Ci-GlyR *gene containing a putative *Ci-GlyR *promoter**. (*A*) A brightfield image partially merged with the GFP expression pattern (red rectangle that corresponds with the view in *C*). (*C, D*) The two image planes on right (*C*) and left (*D*) muscle bands from a typical sample that shows GFP expression in muscle cells and motoneurons (large yellow arrows). Some nerve terminals are branched (red asterisks), while others have long axons that enter the tail nerve cord (small yellow arrowheads not resolvable easily in *I*). *Ciona *muscle bands are composed of three rows of cells, dorsal (D), middle (M), and ventral (V), and are numbered from anterior to posterior. Muscle cells with the highest expression in this specimen are underlined. The background signal in the tunic cells on the surface of the larva is due to autofluorescence that is also present in the controls. Scale bar = 100 μm.

The post-synaptic targets of the putative glycinergic neurons were located by cloning the single copy glycine receptor predicted from the genome sequence (termed Ci-GlyR Additional File 4, and reference [12]). Probes designed for *in situ *hybridization, and antisense incubation for the glycine receptor revealed a pattern of expression in the region of the tail and the MG (Figure 4A, B, C, D,-E, black open and red closed arrowheads, respectively). At least 100 larvae were used for each observation, the figures are representative examples of these patterns. The signals in the tail correspond to the anterior population of developing muscle cells. Co-incubation of the *Ci-GlyR *probe and a *choline acetyltransferase *probe (*ChAT*), revealed co-labeling of cells in the MG (Figure 4F, G), thus positively identifying the *Ci-GlyR *positive cells as motoneurons with their cell bodies located in the MG. To establish better the identity and form of the labeled cells, we cloned a promoter element of 2.5 kb upstream of the *Ci-GlyR *gene that we coupled to a GFP reporter gene. Fertilized eggs were injected with the promoter construct and 18 hours later, fully developed larvae revealed strong GFP labeling of motoneurons in the MG and the muscle cells (Figure 5A, B, C), indicating that both cell types express glycine receptors.

The ligand specificity of Ci-GlyR, was examined by expressing the full length sequence in *Xenopus *oocytes. The receptor is selectively gated by glycine over GABA (Additional File 5A, B). Substitution of ion species in extracellular solutions (Additional File 5C, D) revealed that the reversal potential is sensitive to alterations in chloride concentration, showing that Ci-GlyR is permeant to chloride and likely to be inhibitory.

### Suppression of Ci-GlyR expression

**Figure 6:**
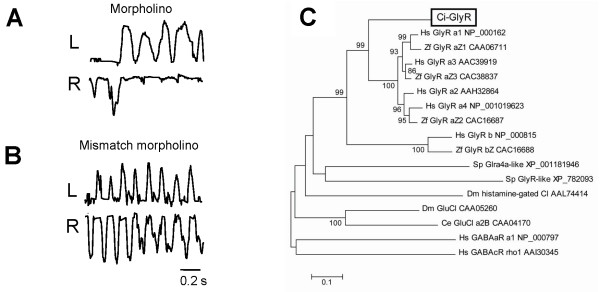
**Function and evolution of Ci-GlyR**. (*A, B*) Effect of gene suppression on larval swimming by morpholino injection into developing eggs. (*A*) Antisense morpholino disrupts alternating swimming in a similar manner to strychnine. (*B*) 5-base mismatched control morpholino is without effect on swimming, larvae are capable of normal alternating swimming (Supp. Video 2). (*C*) The phylogeny of Ci-GlyR compared to other members of the Cys-loop superfamily of ligand gated chloride channels (*Ci-GlyR *is indicated in the box). Hs: *H. sapiens*, Zf: *D. rerio (zebrafish)*, Ci: *C intestinalis*, Ce: *C. elegans*, Dm: *D. melanogaster*, Sp: *S. purpuratus *(sea urchin).

To determine the involvement of the Ci-GlyR in the alternation of tail beats during swimming activity, antisense (+) and 5 base-mismatched (-) morpholino oligonucleotides (MO) were designed and injected into fertilized eggs, and the resulting larvae were assessed phenotypically. 10/10 +MO larvae were unable to swim progressively, and produced series of unilateral tail flicks instead of strict left right alternation (as in the case of the strychnine treated larvae above), and had an otherwise normal phenotype (Figure 6A, Additional File 6). 11/11 -MO larvae swam normally producing alternating tail beats (Figure 6B, Additional File 6). When compared by video analysis there was no significant difference in the left-side swimming frequency between +MO and -MO larvae (13.66 ± 0.01 [n = 10], and 14.20 ± 0.02 Hz [n = 10], respectively). However, there was no organized 'right/left' regular alternation.

### Phylogeny of Ci-GlyR

The phylogeny of Ci-GlyR was examined by comparing available cognate sequences. The protein alignments (Additional File 4) and a phylogenetic tree (Figure 6C) show that Ci-GlyR clusters with vertebrate α-glycine receptors. Although distantly related sequences exist in the sea urchin genome, in non-deuterostome invertebrates no evidence for glycine receptor-like genes was found, and the nearest 'hits' were other ligand-gated ion channel members of the Cys-loop protein superfamily (inhibitory glutamate- and histamine-gated chloride channels).

## Discussion

### Physiology, pharmacology and phylogeny of locomotion

One of the main roles of the nervous system in the ascidian larva is to generate swimming movements that are manifested in alternating tail beats that occur between 10-40 Hz. The motor ganglion (MG) contains around 10 cholinergic motoneurons (4-5 on each side of the midline; [6,18]), and is believed to represent the main motor area controlling swimming. Our microdissection experiments confirm the role of the MG in controlling and generating motor patterns, as 'headless' larvae lacking both otolith and ocellus, but with tail and MG intact, are capable of coordinated swimming movements when L-glutamate is added to the bath. This is reminiscent of the situation in vertebrates where excitatory amino acids are known to induce fictive swimming in isolated vertebrate spinal cords [19]. Thus, a minimal CPG exists in the MG/NC complex.

Our investigations suggest that inhibitory glycinergic transmission is involved in the production of appropriate swimming patterns by providing inhibition of the excitatory activity across the midline, but this function is not necessary for the generation of regular activity on each side. This is based on the findings that strychnine and MO suppression of the glycine receptor do not block pattern generation on either side but disrupted alternation between sides. This is similar to the situation in vertebrates where the effects of block by strychnine could be best explained by a model consisting of elements that are able to convert tonic drive into regular activity with negative coupling across the midline and an additional weak excitatory coupling between both sides [20]. Our results show that rhythmic activity is maintained in the presence of strychnine suggesting that as in vertebrates the tonic drive to the network (glutamate) creates rhythms in the network and that this rhythmic activity is not generated *per se *by coupling between the two sides. It is more difficult to determine if also excitatory cross coupling is present but this seems unlikely as un-coordinated 'writhing' movements were not seen. Finally, we confirm that GABAergic transmission has a modulatory role in controlling swimming rate and duration but has no role in coupling the activity of muscle on either side of the nerve cord [17].

Here we have compared the nature of the ascidian network with the lamprey system because of this animals phylogenetic proximity to lower chordates. It is worth underlining however that some major similarities extend to other simple vertebrate systems such as the frog tadpole. In hatchling frog tadpoles, single motoneurons on either side of the nerve cord produce single spikes (a single action potential per cycle) to drive swimming. At this stage, the cells are not inherently rhythmic though a strictly alternating pattern may be produced by the network. Later in development, the systems matures, neurons develop rhythmicity, and the pattern becomes more complex and flexible [21]. As it is shown here that despite block by strychnine or knockdown by morpholino, activity continues rhythmically on each side, it would seem that the *Ciona *tadpole resembles most closely the mature frog tadpole, which is surprising considering the simplicity of the larval system.

Despite the above similarities, there are also significant (perhaps unique) differences in the ascidian system that are worth pointing out. The finding that the proximal larval muscle cells express glycine receptors was unexpected and indicates that muscle fibers are a more closely integrated part of the network than in vertebrate systems. Indeed, the precise role that these muscle cells play in swimming coordination has yet to be established. It is known that are differences during development in the biophysical properties of muscle cells in pre-hatching and swimming larvae suggesting a period where movements may be driven by myogenic activity [22]. In other invertebrate models such as Drosophila, the neural control of larval crawling has been shown to occur in development after a period of purely myogenic movement [23]. The exact contribution and timing of myogenic *vs*. neutrally driven patterns and their integration has yet to be established in the hatching larva of *Ciona *and could be a promising avenue for future study.

### Location of putative glycinergic interneurons

Our results suggest that at least a population of 2-4 cells recently-identified as 'caudal neurons' or caudal motoneurons' [18,24] that are present in the nerve cord and that lie within the *Hox5 *positive 'spinal' zone [8] may be glycinergic interneurons. This observation has been recently reinforced by the finding that these same caudal neurons express GABA/glycine transporters (VGAT) [24]. It remains to be seen if these cells are a single class of GABA/glycine interneuron or if indeed two separate classes of neurons (GABA and glycine) exist. We would tend to favor the latter hypothesis as the actions of the two neurotransmitters are so different (this paper and [17]. Interestingly, previous studies [24,25] also reported a variation in the number of labeled cells and we also noted a variation in cell numbers between animals. As our experiments were carried out on 3-4 hour hatched larvae, this is unlikely to represent different developmental stages and is likely to be a real (though surprising), animal to animal variation. Future studies should resolve this issue and appropriate molecular markers need to be developed to identify these cells. It is noteworthy that the cell bodies and their processes appear to be confined entirely to the nerve cord, unlike the motoneurons which extend processes along the cord, but also form terminals outside the nerve cord on the muscle cells.

### Glycine receptor function

Molecular markers for the *Ciona *single copy glycine receptor (Ci-GlyR) allowed the potential targets of glycinergic inhibition to be identified. *In situ *hybridization revealed a pattern of expression in the anterior tail muscle and MG neurons. The neural elements were identified as cholinergic motoneurones by double *in situ *hybridization with a *choline acetyltransferase *probe (*ChAT*). In addition, the 2.5 kb sequence upstream of the *Ci-GlyR *gene, presumably containing the promoter for Ci-GlyR, revealed strong GFP labeling of motoneurons in the MG and muscle cells confirming that both cell types express glycine receptors. We interpret these data as indicating that glycine may have a dual role in inhibiting both motoneurons and muscle cells. Further investigation (for example direct electrophysiological recording from the anterior rows of muscle), should help to resolve the issue.

Not surprisingly our results from the expression of the full length sequence in *Xenopus *oocytes showed a very high selectivity for glycine over GABA with a reversal potential sensitive to alterations in chloride concentration. The results indicate that Ci-GlyR is permeant to chloride in a similar way as vertebrate glycine receptors are [26]. The selectivity for glycine over GABA could lead one to speculate that this could be necessary because GABA is active in the system and the short cross-cord distances and the lack of glial sheathing in this system would result in synaptic 'spill over'.

### Phylogeny of Ci-GlyR

The above results also point to the particular features of the phylogeny of Ci-GlyR that we examined by comparing available cognate sequences. The protein alignments and phylogenetic tree show that Ci-GlyR clusters with vertebrate α-glycine receptors. No closely related sequences were found in sea urchin and there were only distant 'hits' with other ligand-gated ion channel members of the Cys-loop protein superfamily (inhibitory glutamate- and histamine-gated chloride channels) in other invertebrates. Until full-sequencing and heterologous expression analysis of sea urchin sequences are carried out, one may conclude tentatively that glycine receptors are likely to be a chordate innovation from an ancestral bilaterian Cys-loop protein, and that they are specifically associated with chordate locomotory networks.

## Conclusions

While the details of the connectivity of motoneurons, muscle and caudal neurons remain to be worked out, these results provide additional evidence to support basal similarities of function between the ascidian MG/NC complex and the vertebrate spinal cord. Thus, it is likely that the role of glycine inhibitory synaptic transmission in controlling coupling of left-right alternation in vertebrate spinal CPGs and ascidians may be traced back to their last common ancestor. The elaborate and cellularly numerous vertebrate network is minimally represented in this ascidian larva by a dramatically reduced number of cells, and not more than 10 moto- and 2-4 inhibitory interneurons that are located in the MC and NC, respectively. These results, along with other homologies between vertebrate and invertebrate chordates, indicate that these animals represent highly relevant models, not only to determine developmental mechanisms, but also to understand the evolution and physiology of the chordate nervous system.

## Authors' contributions

AN, YO and ERB designed the initial experiments, AN, ERB and SP carried out the experiments, and analyzed the data. ERB wrote the paper. AN, YO and SP edited and approved the paper.

## Supplementary Material

Additional file 1 **High speed video clip showing free-swimming, and tethered larval swimming**. Tethered larvae are immobile after microdissection of the foremost part of the 'head', the area containing the motor ganglion remains. Addition of 100 μM glutamate induces regular alternating swimming. Click here for file

Additional file 2 **Effect of picrotoxin on swimming in tethered larvae**. (*A*) Control showing strict left/right (L/R) alternation of tail movements during swimming strokes. (*B*) Phase relation of the autocorrelation on the left side (blue) with the cross correlation (red) between left and right sides. (*C*) The same larvae as *C *after the addition of picrotoxin. (D) Phase relation of the autocorrelation on the left side (blue) with the cross correlation (red) between left and right sides in the presence of picrotoxin. Note that swimming rate and duration increased in picrotoxin Click here for file

Additional file 3 **Glycine immunocytochemistry at the junction of the 'tail' and 'head' region of the ascidian larva**. (*A*) test example with primary antibody, (*B*) control (primary antibody omitted). Note the glycine positive zone in the nerve cord (arrows). Click here for file

Additional file 4 **Alignment of *Ci-GlyR *primary sequence with mammalian glycine receptors**. The well-conserved Cys-loop structures are indicated with lines. Predicted signal peptides and trans-membrane domains are labeled by dotted underlining and shaded rectangles respectively. Click here for file

Additional file 5 **Biophysical properties of Ci-GlyR heterologously expressed in *Xenopus *oocytes at a holding potential of -50 mV**. (*A*) Inward currents generated by application of glycine (30 μM - 3 mM, upper panel) or GABA (10 mM, lower panel). (*B*) Normalized dose-response curve of Ci-GlyR in *Xenopus *oocytes. The EC_50 _of glycine gating of the receptor is estimated to be about 7.5×10^-4 ^M, a similar value to that seen in vertebrate glycine receptors expressed in *Xenopus *oocytes [27]. (*C, D*) Ci-GlyR is selectively permeant to Cl^-^. (*C*) The normalized I-V relationships of Ci-GlyR in the indicated extracellular solutions. Low Cl^- ^(thick curve), but not low Na^+ ^and K^+ ^(gray curve), causes a significant shift in reversal potential. (*D*) Effects of ion substitution on the reversal potential of glycine evoked currents. Small circles show the reversal potential values in replicated experiments (n > 6) and bars at right indicate the mean and SD. NMDG, N-methyl-d-glucamine; MetS, methanesulphonate. Click here for file

Additional file 6 **High speed video clips showing the result of *Ci-GlyR *morpholino on larval swimming**. After (+) *Ci-GlyR *morpholino treatment, larvae are unable to swim in a coordinated way (clip shows two larvae). The third clip shows that after treatment with (-) 5-base mismatched *Ci-GlyR *morpholino, larvae swim normally. Click here for file
