# Supplementary figures and images for: A glycine receptor is involved in the organization of swimming movements in an invertebrate chordate
Source: BMC Neurosci. 2010 Jan 19;11:6. doi: 10.1186/1471-2202-11-6 (PMC2822779; doi:10.1186/1471-2202-11-6)

**A**

Control

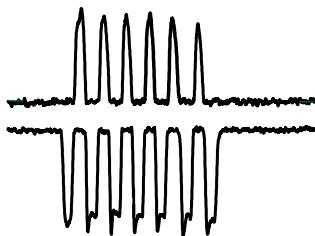**B**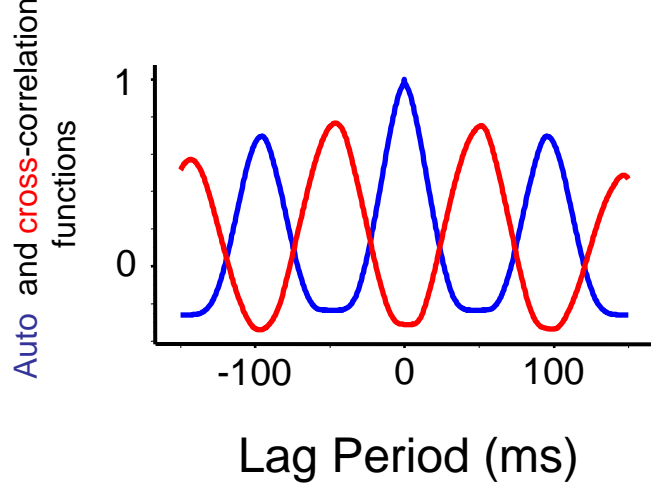**C**10  $\mu$ M picrotoxin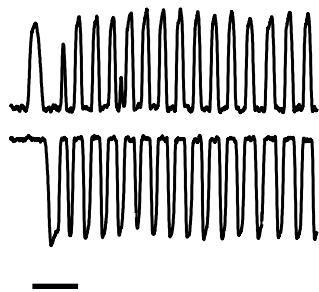

200 ms

**D**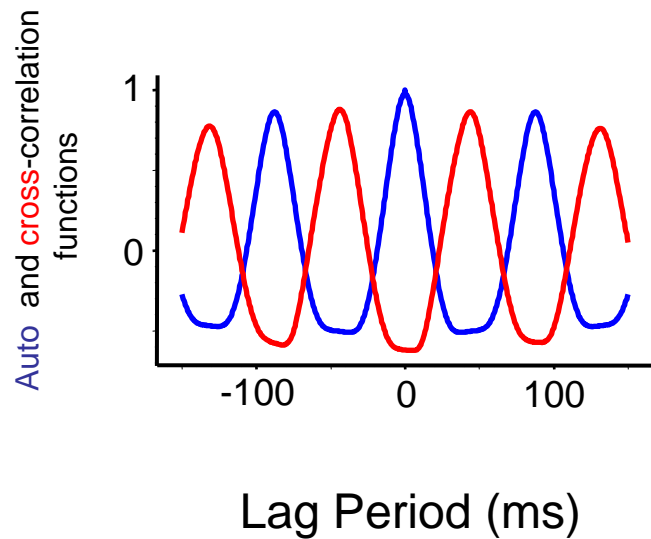

Supplement: Additional file 2 — Effect of picrotoxin on swimming in tethered larvae. (A) Control showing strict left/right (L/R) alternation of tail movements during swimming strokes. (B) Phase relation of the autocorrelation on the left side (blue) with the cross correlation (red) between left and right sides. (C) The same larvae as C after the addition of picrotoxin. (D) Phase relation of the autocorrelation on the left side (blue) with the cross correlation (red) between left and right sides in the presence of picrotoxin. Note that swimming rate and duration increased in picrotoxin [file 1471-2202-11-6-S2.PDF]

**A**

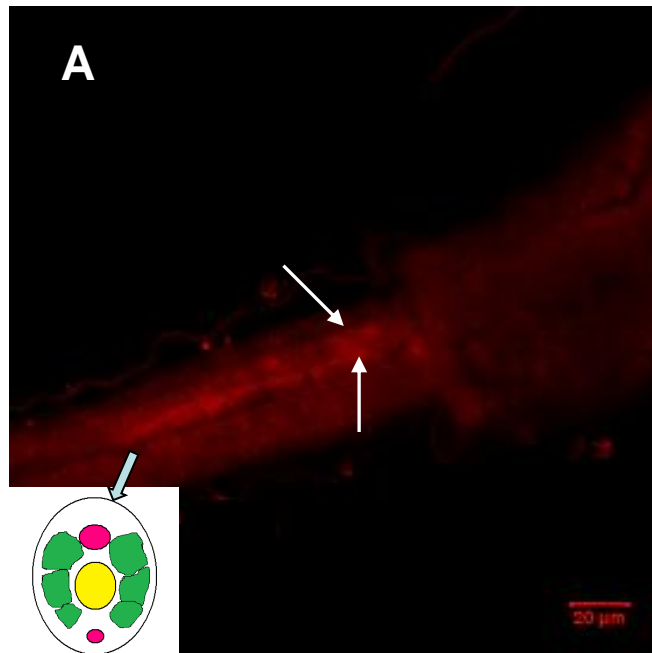

Anti-Gly

**B**

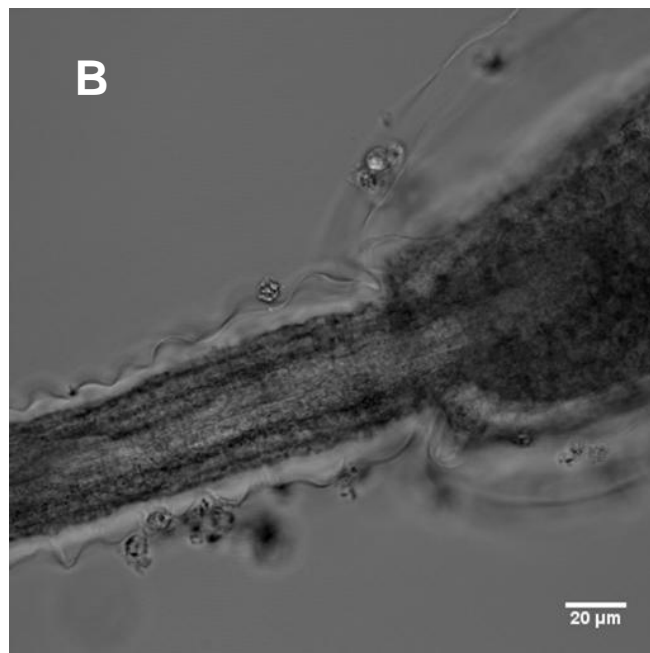

**C**

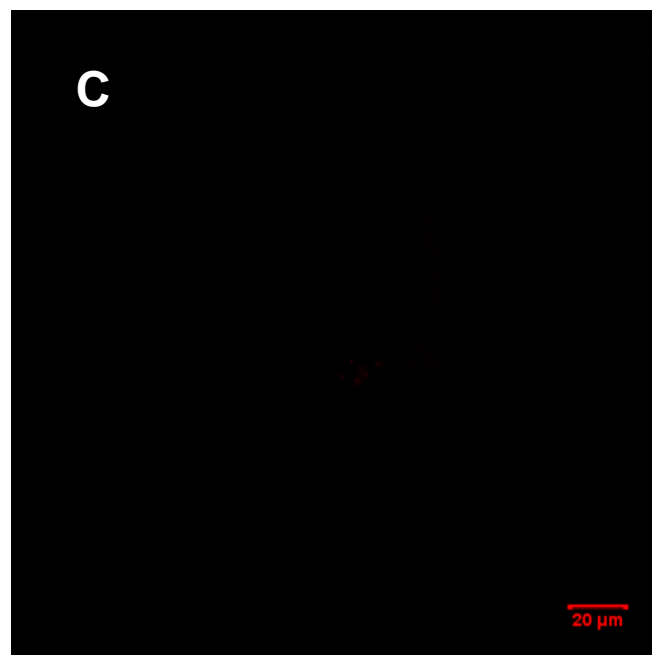

control

**D**

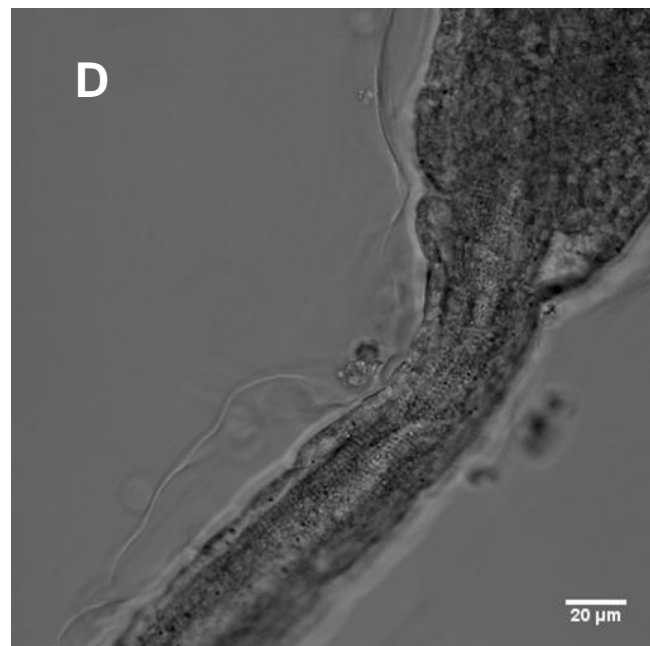

Supplement: Additional file 3 — Glycine immunocytochemistry at the junction of the 'tail' and 'head' region of the ascidian larva. (A) test example with primary antibody, (B) control (primary antibody omitted). Note the glycine positive zone in the nerve cord (arrows). [file 1471-2202-11-6-S3.PDF]

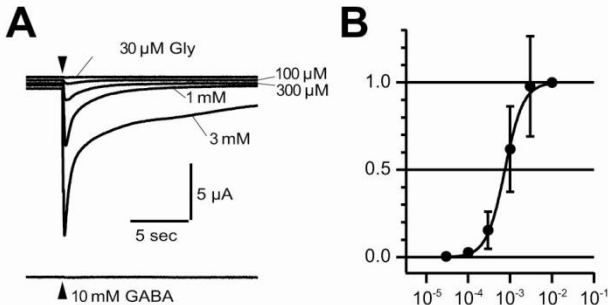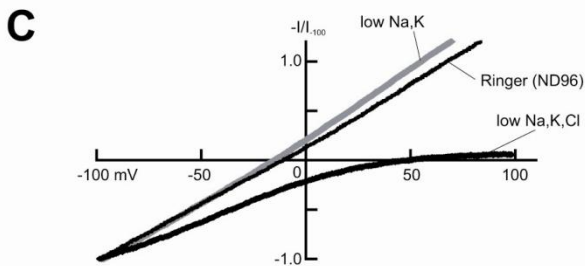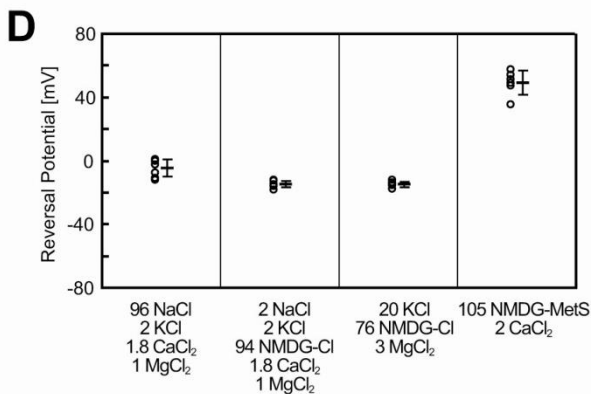

Supplement: Additional file 5 — Biophysical properties of Ci-GlyR heterologously expressed in Xenopus oocytes at a holding potential of -50 mV. (A) Inward currents generated by application of glycine (30 μM - 3 mM, upper panel) or GABA (10 mM, lower panel). (B) Normalized dose-response curve of Ci-GlyR in Xenopus oocytes. The EC50 of glycine gating of the receptor is estimated to be about 7.5×10^-4^ M, a similar value to that seen in vertebrate glycine receptors expressed in Xenopus oocytes [27]. (C, D) Ci-GlyR is selectively permeant to Cl-. (C) The normalized I-V relationships of Ci-GlyR in the indicated extracellular solutions. Low Cl- (thick curve), but not low Na+ and K+ (gray curve), causes a significant shift in reversal potential. (D) Effects of ion substitution on the reversal potential of glycine evoked currents. Small circles show the reversal potential values in replicated experiments (n > 6) and bars at right indicate the mean and SD. NMDG, N-methyl-d-glucamine; MetS, methanesulphonate. [file 1471-2202-11-6-S5.PDF]
