# Supplementary material for: A glycine receptor is involved in the organization of swimming movements in an invertebrate chordate
Source: BMC Neurosci. 2010 Jan 19;11:6. doi: 10.1186/1471-2202-11-6 (PMC2822779; doi:10.1186/1471-2202-11-6)
Supplement: Additional file 4 — Alignment of Ci-GlyR primary sequence with mammalian glycine receptors. The well-conserved Cys-loop structures are indicated with lines. Predicted signal peptides and trans-membrane domains are labeled by dotted underlining and shaded rectangles respectively. [file 1471-2202-11-6-S4.PDF]

```
Ci-GlyR           -MQSQYNVCHFVAVLVLLLCTCILGFDESDNRPLEPRHENLFNAELPIDREYDYDNYYGE
Hs-GlyRa1         ----MYSFNTLR--LYLWETIVFFSLAASKE-----------------------------
Hs-GlyRa2         MNRQLVNILTALFAFFLETNHFRTAFCKDHD-----------------------------
Hs-GlyRa3         -MAHVRHFRTLVSGFYFWEAALLLSLVATKE-----------------------------
Mm-GlyRa4         -MTTLVPASLFLLLWTLPGKVLLSVALAKED-----------------------------
Hs-GlyRb          -MKFLLTTAFLILISLWVEEAYSKEKSSKKGKG---------------------------
                                               .

Ci-GlyR           IPKQRKKTVSTQTHTESTEEKKATYFLDNLFN---GYDQRIRPHHKRSPVNVTVNIFVNS
Hs-GlyRa1         ----------AEAARSAPKPMSPSDFLDKLMGRTSGYDARIRPNFKGPPVNVSCNIFINS
Hs-GlyRa2         ----------SRSGKQPSQTLSPSDFLDKLMGRTSGYDARIRPNFKGPPVNVTCNIFINS
Hs-GlyRa3         ----------TDSARSRSAPMSPSDFLDKLMGRTSGYDARIRPNFKGPPVNVTCNIFINS
Mm-GlyRa4         ----------VKSGLKGSQPMSPSDFLDKLMGRTSGYDARIRPNFKGPPVNVTCNIFINS
Hs-GlyRb          --KKKQYLCPSQQSAEDLARVPANSTSNILNRLLVSYDPRIRPNFKGIPVDVVVNIFINS
                                 .      ..   : *     .** ****:.*  **:*  ***:**

Ci-GlyR           FGSIAETTMDYRVNIFLRCRWNDQRMAF-TGFD-EDAVALHPSMLENIWRPDLFFANEKH
Hs-GlyRa1         FGSIAETTMDYRVNIFLRQQWNDPRLAY-NEYP-DDSLDLDPSMLDSIWKPDLFFANEKG
Hs-GlyRa2         FGSVTETTMDYRVNIFLRQQWNDSRLAY-SEYP-DDSLDLDPSMLDSIWKPDLFFANEKG
Hs-GlyRa3         FGSIAETTMDYRVNIFLRQKWNDPRLAY-SEYP-DDSLDLDPSMLDSIWKPDLFFANEKG
Mm-GlyRa4         FGSVTETTMDYRVNVFLRQQWNDPRLAY-REYP-DDSLDLDPSMLDSIWKPDLFFANEKG
Hs-GlyRb          FGSIQETTMDYRVNIFLRQKWNDPRLKLPSDFRGSDALTVDPTMYKCLWKPDLFFANEKS
                  ***: *********:*** :*** *:     :  .*:: :.*:* .  :*:*********

Ci-GlyR           ANFHEVTTENKLLRIYKNGDVYSSVRLSLTLACAMHLQNFPMDIQTCKMQLESFGYDMRD
Hs-GlyRa1         AHFHEITTDNKLLRISRNGNVLYSIRITLTLACPMDLKNFPMDVQTCIMQLESFGYTMND
Hs-GlyRa2         ANFHDVTTDNKLLRISKNGKVLYSIRLTLTLSCPMDLKNFPMDVQTCTMQLESFGYTMND
Hs-GlyRa3         ANFHEVTTDNKLLRIFKNGNVLYSIRLTLTLSCPMDLKNFPMDVQTCIMQLESFGYTMND
Mm-GlyRa4         ANFHEVTTDNKLLRIFKNGNVLYSIRLTLILSCPMDLKNFPMDIQTCTMQLESFGYTMND
Hs-GlyRb          ANFHDVTQENILLFIFRDGDVLVSMRLSITLSCPLDLTLFPMDTQRCKMQLESFGYTTDD
                  *:**::* :* ** * ::*.*  *:*::: *:*.:.*  **** * * ********   *

Ci-GlyR           LAFQWQEDLP-VQLPPSLTLPQFRIL-GYK-LGSCTKVYN-TGSFTCIEVSFILERQMGY
Hs-GlyRa1         LIFEWQEQGA-VQVADGLTLPQFILK-EEKDLRYCTKHYN-TGKFTCIEARFHLERQMGY
Hs-GlyRa2         LIFEWLSDGP-VQVAEGLTLPQFILK-EEKELGYCTKHYN-TGKFTCIEVKFHLERQMGY
Hs-GlyRa3         LIFEWQDEAP-VQVAEGLTLPQFLLK-EEKDLRYCTKHYN-TGKFTCIEVRFHLERQMGY
Mm-GlyRa4         LMFEWLEDAPAVQVAEGLTLPQFILR-DEKDLGYCTKHYN-TGKFTCIEVKFHLERQMGY
Hs-GlyRb          LRFIWQSGDP-VQLEK-IALPQFDIKKEDIEYGNCTKYYKGTGYYTCVEVIFTLRRQVGF
                  * * * .  . **:   ::**** :         *** *: ** :**:*. * *.**:*:

Ci-GlyR           YVIQTYVPSALIVILSWVSFWINMEAAPARTALGITTVLTMTTQSSGARASLPKVSYVKA
Hs-GlyRa1         YLIQMYIPSLLIVILSWISFWINMDAAPARVGLGITTVLTMTTQSSGSRASLPKVSYVKA
Hs-GlyRa2         YLIQMYIPSLLIVILSWVSFWINMDAAPARVALGITTVLTMTTQSSGSRASLPKVSYVKA
Hs-GlyRa3         YLIQMYIPSLLIVILSWVSFWINMDAAPARVALGITTVLTMTTQSSGSRASLPKVSYVKA
Mm-GlyRa4         YLIQMYIPSLLIVILSWVSFWINMDAAPARVGLGITTVLTMTTQSSGSRASLPKVSYVKA
Hs-GlyRb          YMMGVYAPTLLIVVLSWLSFWINPDASAARVPLGIFSVLSLASECTTLAAELPKVSYVKA
                  *::  * *: ***:***:***** :*:.**. *** :**:::::.:   *.*********

Ci-GlyR           IDTWMAVCLLFVFAALLEFAVVNFLS----RQQQRLIKVNMG------------------
Hs-GlyRa1         IDIWMAVCLLFVFSALLEYAAVNFVS----RQHKELLRFRRKRRHHKE------------
Hs-GlyRa2         IDIWMAVCLLFVFAALLEYAAVNFVS----RQHKEFLRLRRRQKRQNKE-----------
Hs-GlyRa3         IDIWMAVCLLFVFSALLEYAAVNFVS----RQHKELLRFRRKRKNKTEAFALEKFYRFSD
Mm-GlyRa4         IDIWMAVCLLFVFAALLEYAAVNFVS----RQHKEFMRLRRRQRRQRME-----------
Hs-GlyRb          LDVWLIACLLFGFASLVEYAVVQVMLNNPKRVEAEKARIAKAEQADGKGGNVAKKNTVNG
                  :* *: .**** *::*:*:*.*:.:     * . .  :.

Ci-GlyR           --WLIKQKSYRVTEHRSAP-APPSEDSG------------DDATRYCIVGRLPIKEEPPI
Hs-GlyRa1         --DEAGEGRFNFSAYGMGPACLQAKDGI------------SVKGANNSNTTNPPPAPSKS
Hs-GlyRa2         -EDVTRESRFNFSGYGMGH-CLQVKDGT------------AVKA-TPAN-PLPQPP--KD
Hs-GlyRa3         MDDEVRESRFSFTAYGMGP-CLQAKDGM------------TPKGPNHPVQVMP-----KS
Mm-GlyRa4         -EDIIRESRFYFRGYGLGH-CLQARDGG------------PMEG-SSIYSPQPPTPLLKE
Hs-GlyRb          TGTPVHISTLQVGETRCKKVCTSKSDLRSNDFSIVGSLPRDFELSNYDCYGKPIEVNNGL
                             .        .    *                          *

Ci-GlyR           KQSIAEDYKKK----------ALAIDTLSRIIFPTTFLIFNIVYWLSYKIANNDKEFYMK
Hs-GlyRa1         PEEMRKLFIQR----------AKKIDKISRIGFPMAFLIFNMFYWIIYKIVRREDVHNQ-
Hs-GlyRa2         GDAIKKKFVDR----------AKRIDTISRAAFPLAFLIFNIFYWITYKIIRHEDVHKK-
Hs-GlyRa3         PDEMRKVFIDR----------AKKIDTISRACFPLAFLIFNIFYWVIYKILRHEDIHHQQ
Mm-GlyRa4         GETMRKLYVDR----------AKRIDTISRAVFPFTFLVFNIFYWVVYKVLRSEDIHQAL
Hs-GlyRb          GKSQAKNNKKPPPAKPVIPTAAKRIDLYARALFPFCFLFFNVIYWSIYL-----------
                   .   :   .           *  **  :*  **  **.**:.**  *

Ci-GlyR           GAIEYWSD
Hs-GlyRa1         --------
Hs-GlyRa2         --------
Hs-GlyRa3         QD------
Mm-GlyRa4         --------
Hs-GlyRb          --------
```
